# Distribution of electric field in patients with obsessive compulsive disorder treated with deep brain stimulation of the bed nucleus of stria terminalis

**DOI:** 10.1007/s00701-021-04991-0

**Published:** 2021-10-15

**Authors:** Matilda Naesström, Johannes Johansson, Marwan Hariz, Owe Bodlund, Karin Wårdell, Patric Blomstedt

**Affiliations:** 1 Division of Psychiatry, Department of Clinical Sciences, Umeå University, 90187 Umeå, Sweden; 2 Department of Biomedical Engineering, Linköping University, Linköping, Sweden; 3 Unit of Deep Brain Stimulation, Department of Clinical Sciences, Umeå University, Umeå, Sweden; 4 Unit of Functional Neurosurgery, UCL Institute of Neurology, Queen Square, London, UK

**Keywords:** Electric field, **S**imulation, Deep brain stimulation (DBS), Obsessive compulsive disorder (OCD), Bed nucleus of stria terminalis (BNST)

## Abstract

**Background:**

Deep brain stimulation (DBS) is being investigated as a treatment for therapy-refractory obsessive compulsive disorder (OCD). Many different brain targets are being trialled. Several of these targets such as the ventral striatum (including the nucleus accumbens (NAc)), the ventral capsule, the inferior thalamic peduncle, and the bed nucleus of stria terminalis (BNST)) belong to the same network, are anatomically very close to one another, or even overlap. Data is still missing on how various stimulation parameters in a given target will affect surrounding anatomical areas and impact the clinical outcome of DBS.

**Methods:**

In a pilot study of eleven participants with DBS of the BNST, we investigate through patient-specific simulation of electric field, which anatomical areas are affected by the electric field, and if this can be related to the clinical results. Our study combined individual patient’s stimulation parameters at 12- and 24-month follow-up with image data from the preoperative MRI and postoperative CT. These data were used to calculate the distribution of electric field and create individual anatomical models of the field of stimulation.

**Results:**

The individual electric stimulation fields by stimulation in the BNST were similar at both the 12- and 24-month follow-up, involving mainly anterior limb of the internal capsule (ALIC), genu of the internal capsule (IC), BNST, fornix, anteromedial globus pallidus externa (GPe), and the anterior commissure. A statistical significant correlation (*p* < 0.05) between clinical effect measured by the Yale-Brown Obsessive Compulsive Scale and stimulation was found at the 12-month follow-up in the ventral ALIC and anteromedial GPe.

**Conclusions:**

Many of the targets under investigation for OCD are in anatomical proximity. As seen in our study, off-target effects are overlapping. Therefore, DBS in the region of ALIC, NAc, and BNST may perhaps be considered to be stimulation of the same target.

## Introduction

Obsessive compulsive disorder (OCD) is a chronic condition driven by intrusive anxiety-provoking thoughts (obsessions) that lead to repetitive behaviour (compulsions) to alleviate anxiety. The most common model for the pathology in OCD is dysregulation in cortico-striato-thalamo-cortical (CSTC) networks [49]. The prevalence of OCD is around 2%, and about 10% of affected patients suffer from severe symptoms, despite best practice pharmacological and psychotherapeutic treatment [14]. Therefore, other treatment options are being investigated for therapy-refractory OCD, including deep brain stimulation (DBS) [45].

DBS is an established treatment for movement disorders [27]. Since DBS for treatment-refractory OCD was first suggested by Nuttin et al. (1999), around ten different brain targets have been investigated [1, 12, 15, 19, 24, 29, 31, 33, 41–43, 47, 55, 56]. Most of these targets are located subcortically in and surrounding the basal ganglia. The basal ganglia is a constellation of deeply located nuclei in the for- and midbrain. The primary role of the basal ganglia is to synchronise behaviour in an integrated way in a given situation. As part of this, the basal ganglia are involved in several sensory, motor, cognitive, and emotional functions, maintained by being a part of CSTC networks [59]. Also associated with the CSTC network is the anterior limb of the internal capsule (ALIC), the first target introduced for DBS for OCD [47]. The ALIC is an important network communicator between many regions involved in cognitive and emotional processes, including the pre-frontal cortex and the striatum [18].

Both the optimal target and the mechanism of action of DBS in these targets are still unknown. Apart from belonging to the same network, several of these targets such as the ventral striatum (including the nucleus accumbens, (NAc)), the ALIC, the inferior thalamic peduncle (ITP), and the bed nucleus of stria terminalis (BNST) are anatomically very close to one another or even overlap [26]. Additionally, data is still missing on how various stimulation parameters in a given target will affect surrounding anatomical areas and impact the clinical outcome of DBS [4, 6]. There are suggestions that anatomical areas targeted by stimulation overlap [51]. Data on which regions are affected by the stimulation in these targets could be helpful to pool evidence on safety and efficacy.

It is possible to estimate the affected field around the active DBS contacts by patient-specific finite element method simulations or simpler electric field models. Such estimates have, for example, been used to study the optimal placement of DBS leads in patients with Gilles de la Tourette syndrome and essential tremor [2, 13]. Here, in a pilot study of DBS in the bed nucleus of stria terminalis for OCD, we investigate through patient-specific simulation of the electric field which anatomical areas are affected by the electric field and if this can be related to the clinical results.

## Methods

### Patients

Eleven consecutive patients were included in this study (7 females, age 21–59). Their Yale-Brown Obsessive Compulsive Scale (Y-BOCS) score ranged between 29 and 38. The disease duration ranged between 5 and 46 years, and all patients had failed previous pharmacotherapy and cognitive behavioural therapy (CBT) trials. The study was approved by the regional ethical board of the Umeå University Hospital (No. 08-090 M). Clinical results at 12-month follow-up from this pilot study have previously been reported [46].

### Surgical procedure

**Fig. 1:**
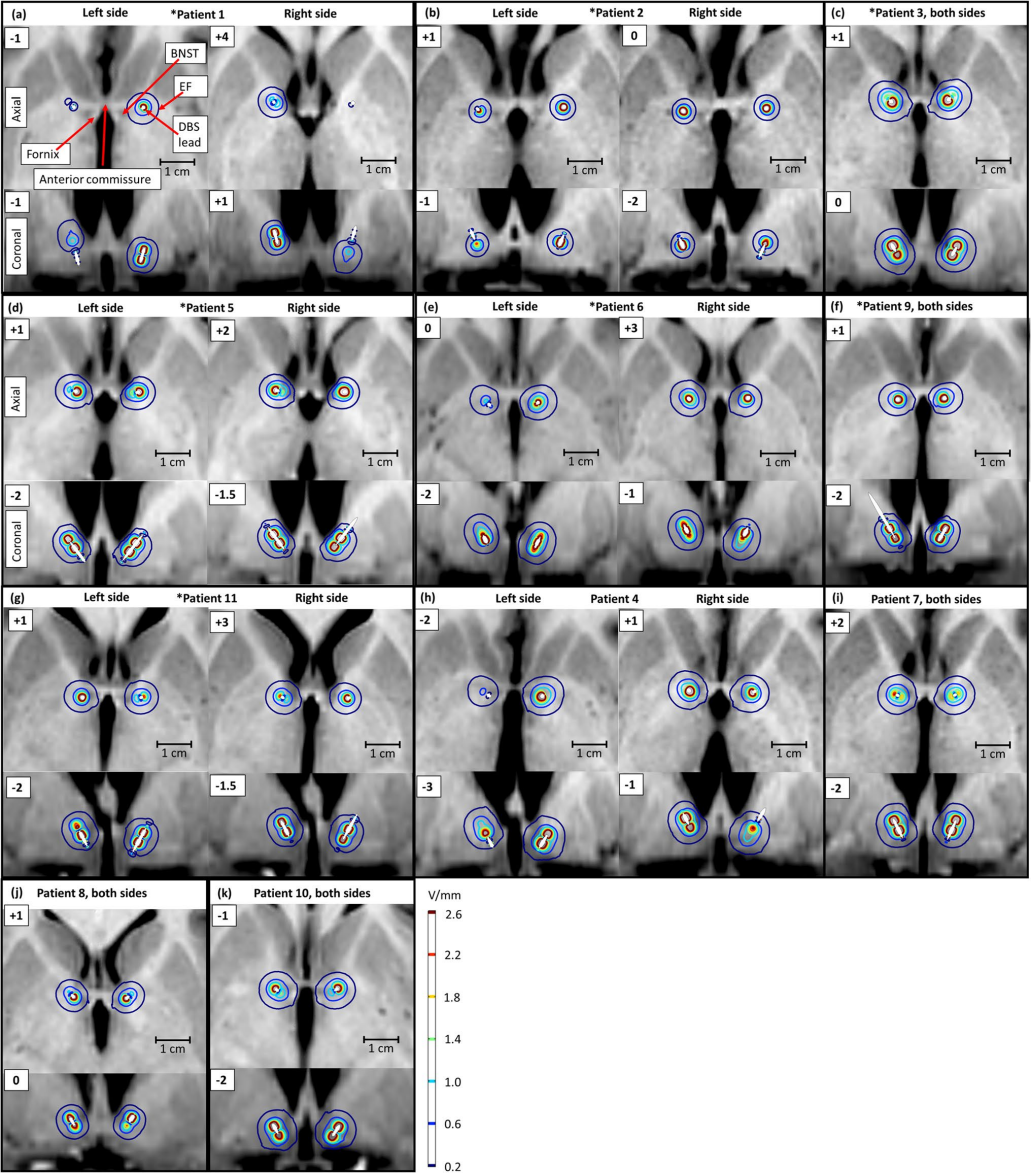
The encircled areas show individual electric fields at 12 months’ follow-up in each of the 11 patients

**Fig. 2:**
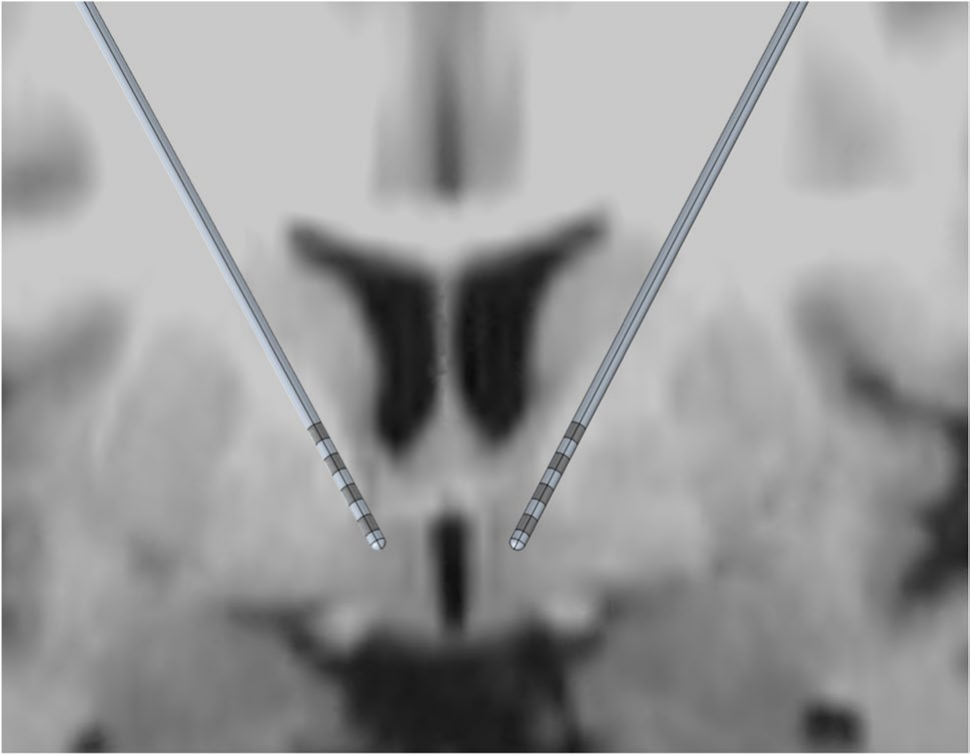
Finite element method model of two Medtronics 3387 leads visualized along the plane of the electrodes. The deepest contact lies 3 mm below the AC-PC plane

The surgery was performed with the Leksell stereotactic frame in general anaesthesia. Stereotactic imaging was done on a Philips Achieva dStream 1.5-T MR machine using T2- and volumetric T1-weighted sequences with an image resolution of 1 × 1 × 2 mm. On the stereotactic T2-weighted MRI, the BNST was visually identified on thin slice axial scans, posterior to the anterior commissure, and lateral to the fornix at the level of the anterior commissure-posterior commissure (AC-PC) line (Fig. 1). Calculating target coordinates and trajectories were done using FrameLink/Stealth Cranial (Medtronic, Minneapolis, USA). An entry point for the lead trajectory was chosen 35–50 mm lateral to the midline and about 0–15 mm anterior to the coronal suture to provide a trajectory intubating the ventral part of the ALIC. The target point for the deepest contact was chosen 3 mm below the AC-PC plane (Fig. 2) [54]. The quadripolar electrodes (Medtronic, model 3387 or 3389) were connected to an implantable pulse generator (Medtronic, PC) in the sub-clavicular area during the same surgical session. To verify the lead location, a postoperative CT was done on a GE LightSpeed VCT machine with an image resolution between 0.43–0.59 × 0.43–0.59 × 1.25 mm. The CT image was then fused with the preoperative stereotactic MRI.

### Programming and post-operative follow-up

Stimulation was typically started 12 (range 3–30) days after surgery. The initial programming session consisted of a screening of each individual electrode contact, mainly for side effects. During the following months, stimulation voltage was further increased to reduce obsessions, compulsions, and anxiety. In case of side effects, the programming was reverted to lower voltage settings, and the titration was more gradual. Table 1 shows individual chronic stimulation parameters at 12 and 24 months after the surgery.

**Table 1:** Stimulation settings and % improvement in Y-BOCS at 12 and 24 months follow up

		12 months					24 months				
Patient no	Electrode	Contacts	Volt	Pulse width (msec)	Frequency (Hz)	Y-BOCS improvement %	Contacts	Volt	Pulse width (msec)	Frequency (Hz)	Y-BOCS improvement %
1	3387	Left: − 0, − 1 Right: − 10, − 11	3.2	90	130	53%	Left: − 0, − 1 Right: − 9,10 − 11	5.2/ 4.5	90	130	40%
2	3389	Left: − 2 Right: − 9	3.5	60	130	56%	Left: + 0, − 1 − 2, + 3 Right: − 8, − 9 + 10	3.5	150	130	66%
3	3387	Left: − 1, − 2 Right: − 9, − 10	5	120	130	60%	Left: − 1, − 2 Right: − 9, − 10	4.4	120	130	49%
4	3387	Left: − 1, − 2 Right: − 9, − 10	4.2	150	130	10%	Left: − 1, − 2 Right: − 9, − 10	4.2	120	130	38%
5	3387	Left: − 1, − 2 Right: − 9, − 10	4.1	90	130	45%	Left: − 1, − 2 Right: − 9, − 10	5	90	130	42%
6	3389	Left: − 0, − 1 − 2, Right: − 9 − 10, − 11	4	60	130	47%	Left: − 0, − 1 − 2, Right: − 9 − 10, − 11	4	60	130	13%
7	3387	Left: − 1, − 2 Right: − 9, − 10	4.9	90	130	34%	Left: − 1, − 2 Right: − 9, − 10	5.1	90	130	34%
8	3387	Left: − 1, − 2	3.8	60	130	25%	Left: − 1, − 2	3.8	60	130	34%
9	3387	Left: − 1, − 2 Right: − 9, − 10	4.4	60	130	41%	Left: − 1, − 2 Right: − 9, − 10	4.4	60	130	65%
10	3387	Left: − 1, − 2 Right: − 9, − 10	4.3	90	130	25%	Left: − 1, − 2 Right: − 9, − 10	3.9	90	130	22%
11	3387	Left: − 1, − 2 Right: − 9, − 10	3.5	90	130	36%	Left: − 1, − 2 Right: − 9, − 10	3.5	90	130	33%

### Computer simulations of electric field

Models of the Medtronic leads were made in Comsol Multiphysics 5.3a (Comsol AB, Sweden). The electric field magnitude (*EF)* was calculated from the equation for steady currents, which depends on the tissue-dependent electric conductivity. An in-house developed software (ELMA) [35] was used to classify the tissue into grey matter, white matter and cerebrospinal fluid based on the preoperative T1-weighted MRI of each individual patient [4, 6]. The electric conductivity was assigned according to tissue type from tabulated values [3, 22] weighted with the spectral distribution of the DBS pulse shape [57]. The active cathode contacts were assigned the electric potential used for the individual patient while the surrounding surfaces were set to ground. For details of the modelling and simulation, see [4]. Tissue within a threshold electric field magnitude (*EF*_t_) was assumed to be activated by the stimulation. This threshold is pulse width dependent with lower *EF*_t_ for longer pulse widths [23], giving a similar effect from increasing pulse width as from increasing the voltage. *EF*_t_ has been estimated to be 0.20 V/mm at the pulse width of 60 µs to 0.14 V/mm at the pulse width of 150 µs based on experimental studies by Alexis Kuncel et al. [38] and Mario Rizzone et al. [52]. For details, see reference [34]. This threshold has been used in several previous studies [4, 7, 28]. The electrodes were aligned by their artefacts in the postoperative CT that had been linearly co-registered with the preoperative MRI (FLIRT, FSL [32]). Meshes of approximately 800,000 tetrahedral elements were used for the simulations, and the volumes within the activation threshold *EF*_t_ were exported as logical matrices with voxels corresponding to the voxels of the preoperative MRI. Pulse width–adjusted *EF* were also exported in the same voxels with an adjustment factor of 0.2/0.14 = 1.43 for 150-µs pulses. For each patient, a region of interest (ROI) 80 × 60 × 30 mm centred on the anterior commissure was selected for the export.

### Stimulation analysis and statistics

The sections of the preoperative T1 images in the ROI exported region were non-linearly co-registered (ANTs [9]), which produces an averaged template image of all patients and individual transformation matrices to it. These transformation matrices were applied to the activated tissue matrices in order to transform them to the averaged template geometry. For each voxel in the template geometry, a linear regression analysis (Matlab, Mathworks, USA) was performed between pulse width-adjusted electric field strength above *EF*_t_ and the % change at 12 and 24 months in Y-BOCS scores. A permutation test for type I errors for multiple comparisons was performed according to a method described by Eisenstein et al. [17]. The voxels with a positive correlation at a significance level of *p* < 0.05 were stored for each parameter. The voxels activated in the simulations in at least one patient with an improvement of at least 35% were also stored for each parameter. The results were visualized in 3D Slicer 4.6.2 [20].

## Results

### Stimulation parameters

All patients had monopolar stimulation (Table 1 shows details of programming settings for individual patients). Mean ± SD stimulation parameters at 12 months were 4.2 ± 0.5 V, pulse width 87 ± 28 µs, and frequency 130 ± 0 Hz. For the seven responders, the mean ± SD stimulation parameters were 4 ± 0.6 V, pulse width 81 ± 23 µs, and frequency 130 ± 0 Hz. For the four non-responders, the mean ± SD stimulation parameters were 4.3 ± 0.5 V, pulse width 98 ± 38 µs, and frequency 130 ± 0 Hz.

Mean ± SD parameters at 24 months were 4.2 ± 0.6 V, pulse width 93 ± 28 µs, and frequency 130 ± 0 Hz. For the six responders, the mean ± SD stimulation parameters were 4.4 ± 0.5 V, pulse width 105 ± 31 µs, and frequency 130 ± 0 Hz. For the five non-responders, the mean ± SD stimulation parameters were 4.1 ± 0.6 V, pulse width 78 ± 16 µs, and frequency 130 ± 0 Hz. (Table 1).

The individual electric stimulation fields, with some individual variances, involved the ALIC, the genu of IC, the ventral part of the caudate including parts of the nucleus accumbens, the BNST and touching the fornix, the anteromedial putamen, and the anterior globus pallidus externa (GPe) and interna (GPi) (Fig. 1). The electric field, on average, extended more lateral and anterior into the GPe on both sides for the responders.

At 24-month follow-up, the individual stimulation fields, with some individual variances, involved the ALIC, the genu of IC, the ventral caudate nucleus including parts of the nucleus accumbens, the fornix, the BNST, and touching the GPe and GPi. There were no significant visual differences of affected anatomical targets between 12 and 24 months in the individual stimulation fields.

### Correlation between electric stimulation field and clinical effects on Y-BOCS

**Fig. 3:**
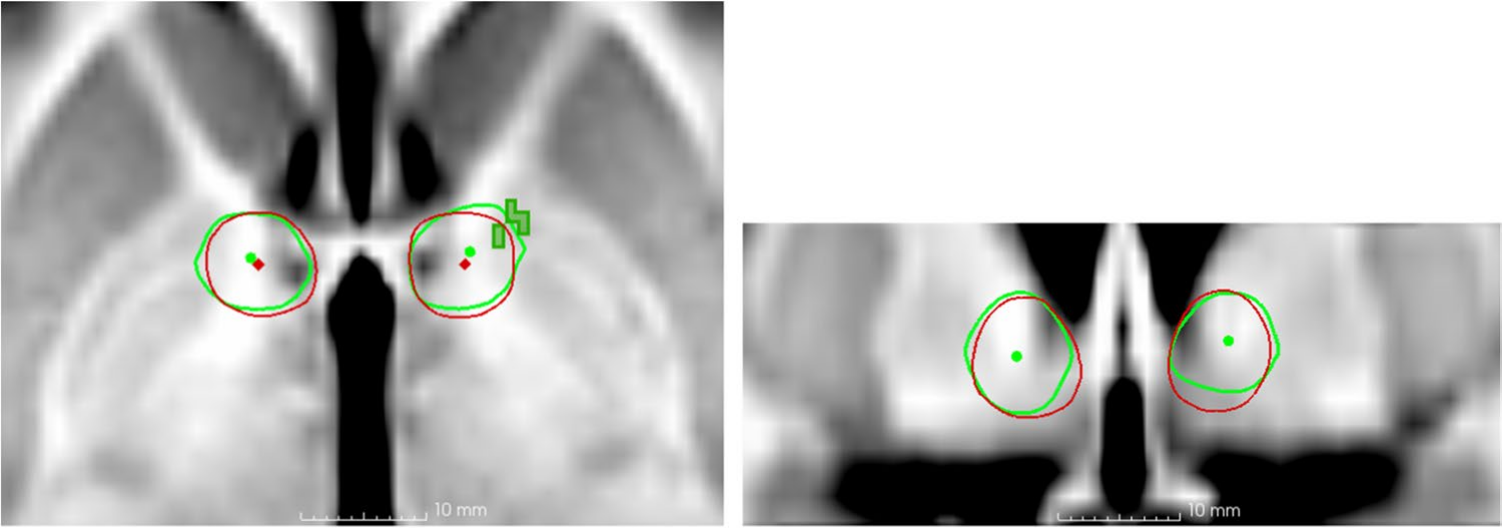
Group simulation fields at 12 months’ follow-up

Stimulation areas in responders involved the ALIC, genu of IC, AC, BNST, fornix, GPe, GPi and touching onto the ventral part of the head of caudate nucleus. Statistically significant results between voxel-based stimulation area and clinical effect of Y-BOCS reduction (*p* < 0.05) was found in the ALIC and anteromedial GPe (dark green voxels in Fig. 3), but the permutation test showed that these were not strong enough to discount type I errors.

At 24-month follow-up, the area of stimulation for responders was almost the same as at 12 months, involving mainly ALIC, genu of IC, AC, BNST, fornix, GPe, GPi and touching onto the most ventral part of the head of caudate nucleus. There were no longer any significant results with linear regression analysis between stimulation area and clinical effect on Y-BOCS reduction.

## Discussion

In our study, a statistically significant correlation was found between clinical effect at 12 months and stimulation field in the ventral ALIC and anteromedial GPe. To the best of our knowledge, this is the first report on the distribution of electric fields in this location for DBS in OCD.

In the literature, there are so far ten different brain targets suggested for OCD: anterior dorsal internal capsule, ALIC, nucleus accumbens, anteromedial subthalamic nucleus, medial forebrain bundle, BNST, caudate nucleus, ITP, dorsomedial, and ventral anterior nucleus of the thalamus [37, 56].

The ALIC is historically a well-studied and used target for capsulotomies in OCD and was therefore selected as the target for the first reported DBS study in OCD [47]. The ALIC is also the most studied target for DBS in OCD, with the largest cohort reported by Denys et al. with 70 participants [16].

The BNST has been suggested as a possible target for DBS in severe OCD [48]. This centrally located nucleus has vast connections with many limbic-related networks, and dysfunction in these pathways is believed to have an important role in anxiety disorders, such as OCD [11, 39]. A few clinical studies, including two randomized trials, have been published demonstrating an effect on obsessions, compulsions, and associated anxiety and depressive symptoms in this target [19, 31, 41, 44, 46, 50].

**Fig. 4:**
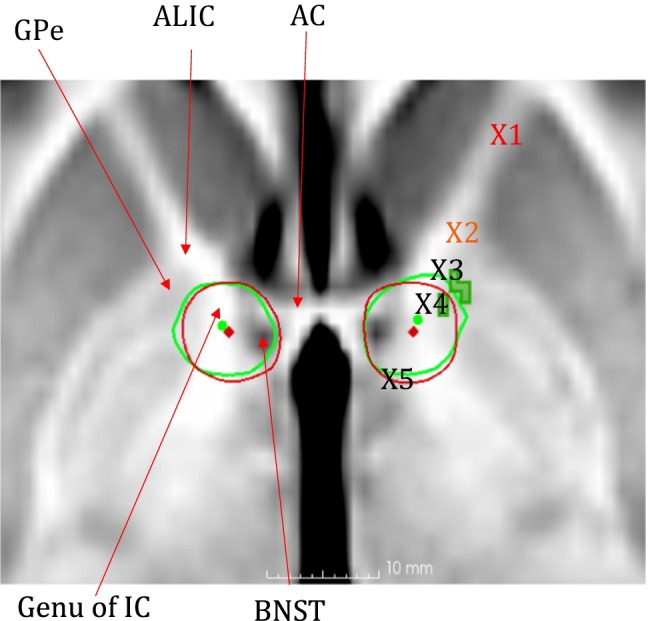
Anatomical target overview

Several of the targets under investigation for OCD are anatomically near the BNST and can be found within this field of stimulation, as illustrated in Fig. 4. That is, the ALIC where DBS for OCD was first suggested by Nuttin et al. (1999) and the posterior location of the IC towards the BNST as suggested by Greenberg et al. (2010) [25, 47]. In the worldwide multicentre study from Greenberg et al. in 2010, the authors described limited effect and high stimulation parameters needed in the more anterior targets (marked as X1 and X2 in Fig. 4) [25]. One of the anterior targets had a better effect (marked as X3 in Fig. 4). However, this target required much higher stimulation parameters to achieve similar results as in the more posterior position (marked as X4 in Fig. 4). A better result in more posterior locations was also confirmed later in studies by Munckhof et al. (2013) and Tyagi et al. (2019) [25, 56, 58]. Jimenez-Ponce described a good response in 5 participants with DBS in the ITP target marked as X5 in the same illustration, however, with very high stimulation settings of 5.0 V and pulse width 450 µs [33].

In our study, the field of stimulation the BNST were similar at both the 12- and 24-month follow-up involving mainly ALIC, genu of IC, BNST, fornix, AC, and anteromedial GPe. In the group simulation fields at 12-month follow-up, we found a positive correlation between clinical improvement and electric field strength in the GPe on the left side. It can also be seen that the electric field extends more lateral and anterior into the GPe on both sides for the responders on the individual simulation models. The GPe is part of the CSTC network, in which dysregulation is the most common model for pathology in OCD [53]. Especially the anteromedial part of the GPe has been suggested as crucial for obsessional compulsive symptoms [21]. Fibres passing through or near the GPe may be involved in the clinical effects of DBS for OCD. However, the statistical significance for the region could not be shown when adjusting for multiple comparisons. The results should thus be taken with great caution. The main fibres involved in OCD affected by the DBS probably go from the thalamus through the genu and anterior limb of the internal capsule along X3-X5 in Fig. 4. DBS in the targets along this line, including the BNST, seem to give satisfactory clinical results in OCD [25, 33, 46]. Further, stimulation delivered to one area has potential off-target effects. Hence, there is a probability that DBS in targets such as the NAc, ALIC, the ventral part of IC, the ITP and BNST will affect neighbouring structures/targets due to the large field of stimulation in this small anatomical region.

Further, since the clinical effects of DBS with all probability do not stem from a single point but from networks, similar effects can probably be achieved by stimulation at different points within these networks [10, 30]. In conclusion, many of these targets for OCD are in anatomical proximity, and as we demonstrated in our study, off-target effects overlap. Therefore, DBS in the region of ALIC, NAc, and BNST may perhaps be considered to be a stimulation of the same target. This off-target effect could explain why Alonso et al. found no demonstrable differences between DBS in ALIC, NAc, and DBS in the BNST [5]. Raviv et al. have also pointed out the inconsistency of target descriptions and nomenclature in the literature and the often marginal difference between different targets, especially with consideration to off-target effects of stimulation [51]. Therefore, they suggested standardizing the nomenclature and defining the targets ALIC, BNST, NAc, VC/VS as “striatal region”.

Since it is well known from DBS in movement disorders that different targets can be used for the same condition, and since this seems to be the case also in OCD, perhaps a more pressing issue is why DBS for OCD seems to have such varying results in different patients. In our material, the electrode placement and anatomical stimulation field were very similar between responders and non-responders. Hence, there should be other factors that contribute to this difference in response that cannot be identified by just studying the electric fields. Which factors do or do not contribute to response-prediction in DBS for OCD is much-needed knowledge.

It can be noted that the electric field threshold level of 0.2 V/mm has been estimated to correspond to the activation of axons with an outer diameter of 3.4 µm [8]. Such large axons are relatively uncommon in the brain, and smaller axons require a stronger electric field for activation [8, 36, 40]. The effect of the DBS is thus expected to be stronger closer to the active contact where the electric field is stronger. This is the reason the voxel-wise statistics were done with linear regression between *EF* and clinical effect rather than just between activated voxels and clinical effect as was done in, e.g. Akbarian-Tefaghi et al. [2].

## Limitations

The main limitation of our study is the small number of patients. Electrodes were similarly placed, obtaining statistically significant comparisons for lead and electric field locations difficult.

## Conclusion

This study analysed the distribution of electric fields in participants treated with DBS in the BNST. We found that the fields of stimulation in the BNST were similar at the 12- and 24-month follow-up, involving mainly ALIC, genu of IC, BNST, fornix, anteromedial GPe, and the AC. Many of the targets under investigation in DBS for OCD are in anatomical proximity, and as we demonstrated in our study, off-target effects are overlapping. Therefore, DBS in the region of ALIC, NAc and BNST may perhaps be considered to be a stimulation of the same target.

## Abbreviations

AC-PC: Anterior commissure-posterior commissure
ALIC: Anterior limb of internal capsule
BNST: Bed nucleus of stria terminalis
CBT: Cognitive behavioural therapy
CT: Computed tomography
DBS: Deep brain stimulation
EF: Electric field magnitude
GPe: Globus pallidus externa
GPi: Globus pallidus interna
IC: Internal capsule
ITP: Inferior thalamic peduncle
MR: Magnetic resonance
NAc: Nucleus accumbens
OCD: Obsessive compulsive-disorder
ROI: Region of interest
SD: Standard deviation
Y-BOCS: Yale-Brown Obsessive Compulsive Scale
